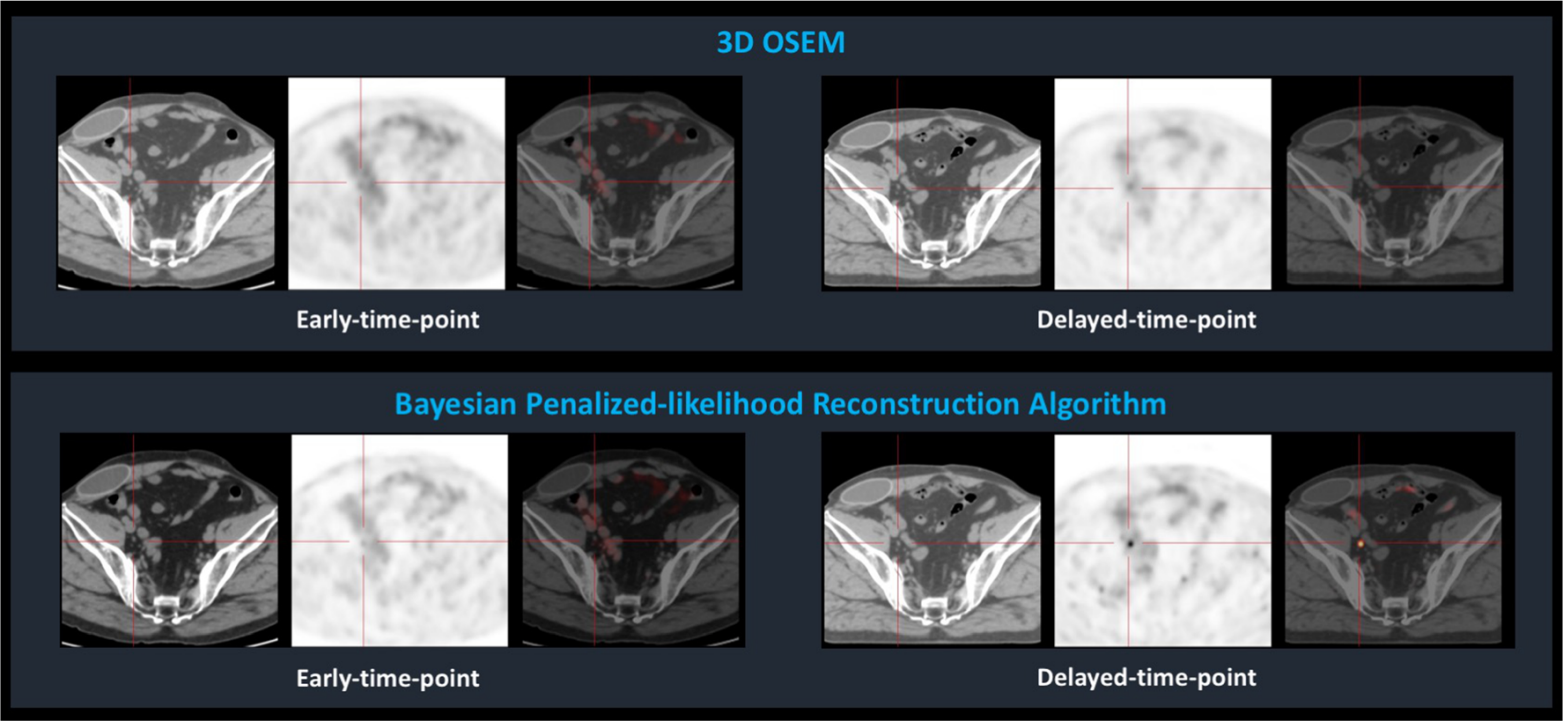# The impact of a Bayesian penalized-likelihood reconstruction algorithm on delayed-time-point Ga-68-PSMA PET for improved recurrent prostate cancer detection

**DOI:** 10.1007/s00259-018-4023-2

**Published:** 2018-04-20

**Authors:** Tiago Sampaio Vieira, Diogo Borges Faria, Fernando Azevedo Silva, Sérgio Barroso, Graça Fonseca, José Pereira Oliveira

**Affiliations:** 1 HPP - Medicina Molecular SA, Lenitudes Medical Center & Research, Apartado 1064, EC Pedro Hispano, 4101-001 Porto, Portugal; 2 HPP - Medicina Molecular SA; Lenitudes Medical Center & Research; School of Health Sciences, University of Aveiro, Aveiro, Portugal; 3 Júlio Teixeira SA - Radioterapia, Porto, Portugal

The benefit of using Ga-68-PSMA PET for patients with biochemical recurrence after radical prostatectomy is high [1, 2], and a delayed-time-point (DTP) acquisition protocol has proved to improve the capacity of Ga-68-PSMA PET to detect prostate cancer metastases [3]. New hardware and reconstruction methods are challenging the resolution limits of PET imaging, and Bayesian penalized-likelihood reconstruction algorithms (BPLA) are paradigmatic of this ongoing revolution. BPLA allows an effective convergence, in contrast to Ordered Subset Expectation Maximization (OSEM), which must be stopped before contrast convergence to prevent excessive image noise [4]. We present the Ga-68-PSMA PET images of a man with biochemical recurrence of prostate cancer (PSA = 0.51 ng/ml). A dual-time-point acquisition protocol (1 h and 2 h after radiopharmaceutical administration) was conducted in a Discovery IQ4R, and images were reconstructed using OSEM and BPLA. Early-time-point PET did not reveal foci of abnormally increased uptake, either using OSEM or BPLA. DTP images revealed a focus of increased uptake in a 3 mm right external iliac lymph node when using BPLA, not visible with OSEM. After robotic radiosurgery, PSA values decreased to undetectable, confirming the focus as a metastasis. Ga-68-PSMA PET is advantageous over other imaging techniques to detect prostate cancer recurrence, especially in patients with low PSA levels, but the reported detection rate of metastatic sites in patients with biochemical recurrence after radical prostatectomy in a PSA-range of 0.5–1.0 ng/ml does not surpass 73% [5]. Applying a BPLA to a DTP protocol may further improve the detection rate of Ga-68-PSMA PET for recurrent prostate cancer.